# Interview with a Retrovirologist: Sebla B. Kutluay in conversation with Carol Carter

**DOI:** 10.1186/s12977-021-00562-4

**Published:** 2021-07-01

**Authors:** Carol Carter, Sebla B. Kutluay

**Affiliations:** 1 Microbiology & Immunology, Stony Brook University, Life Sciences Bldg Rm 248, Stony Brook, NY 11794-5222 USA; 2 Department of Molecular Microbiology, Washington University School of Medicine, 660 S. Euclid Avenue, Campus Box 8230, St. Louis, MO 63110 USA

*Retrovirology is pleased to inaugurate a regular segment called “Interview with a Retrovirologist”, in which two scientists discuss their careers, with the goal of highlighting leaders and rising stars, celebrating diversity and inspiring the next generation of scientists. We are thrilled that our first pair of scientists is Drs. Carol Carter, Professor of Microbiology and Immunology, SUNY Stony Brook and Sebla Kutluay, Assistant Professor of Molecular Microbiology, Washington University, St. Louis. We learned a lot about both Carol and Sebla from this piece, and hope that the readers of Retrovirology find it equally thought-provoking.*

## Carol Carter: Biography

**Fig. 1:**
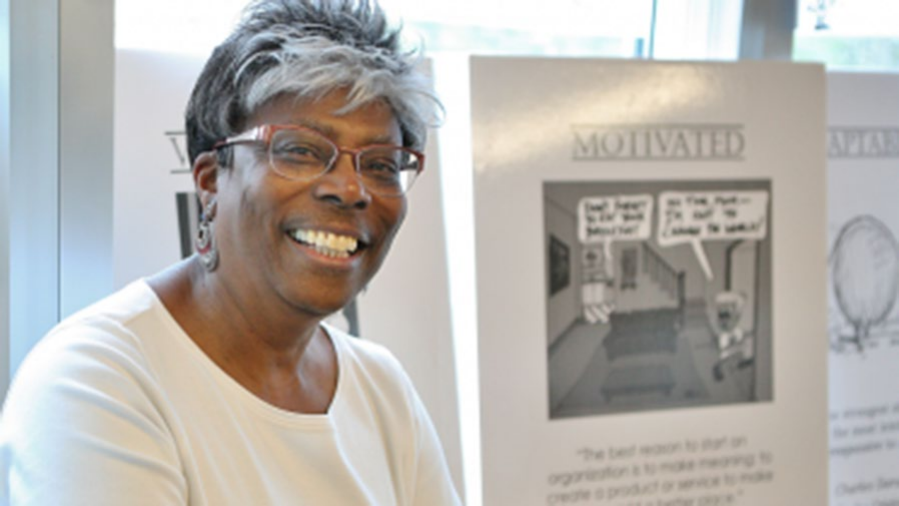
Carter bio photo

Carol Carter, PhD, is a professor in the Department of Microbiology and Immunology at Stony Brook University Renaissance School of Medicine (Fig. 1). She was born and raised in Harlem, New York City, NY and graduated from City College of New York, which is said to have produced more “embryo PhDs” than any other public institution and currently placed in the top 1.2% of universities worldwide in terms of academic excellence by the Center for world University Rankings. She obtained her PhD at Yale University under the mentorship of Drs. Francis Black and Ann Schluederberg studying measles virus replication and pursued postdoctoral studies on reovirus in the laboratory of Dr. Aaron Shatkin at the Roche Institute of Molecular Biology. She established her independent career at Stony Brook University in 1975 and entered the world of HIV/AIDS research in the 1980’s where she has contributed to understanding of retroviral protease activation, capsid structure and assembly and engagement of Tsg101 and ESCRT machinery, in hopes of translating bench observations to antiviral drug development. To “Next Gen”(eration) researchers she notes that being both a gender and ethnic "double minority" in academic research brings with it both overt and covert hurdles and challenges but also provides a unique opportunity to attain a network of friends and supporters who are highly diverse in gender, ethnicity and geographical origin. She believes they provide her with perspectives that broaden her both scientifically and personally.

## Sebla B. Kutluay: Biography

**Fig. 2:**
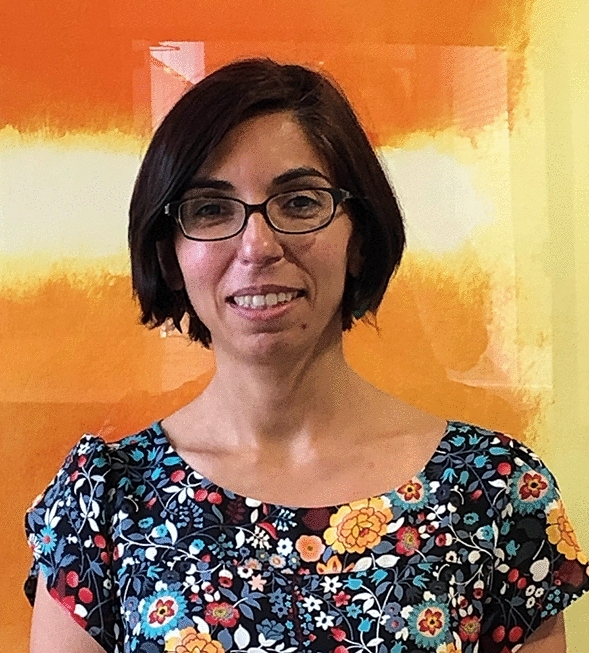
Kutluay bio photo

Sebla B. Kutluay, PhD, is an assistant professor in the Department of Molecular Microbiology at Washington University School of Medicine (Fig. 2). She was born and raised in Turkey. After completing her undergraduate studies in Turkey, she obtained her PhD at Michigan State University under Dr. Steven J. Trizenberg’s mentorship studying chromatin regulation of herpes simplex virus genomic DNA during lytic and latent infections. Dr. Kutluay conducted her postdoctoral studies in the lab of Dr. Paul Bieniasz at Rockefeller University, where she made seminal discoveries in HIV-1 particle assembly, maturation, selective genome packaging and virus-host interactions. She established her independent group in 2015 and continues to study how several viral and host RNA-binding proteins regulate HIV-1, and more recently SARS-CoV-2 replication.

*SK: Such a pleasure to be having this inaugural interview series with you today. Let’s start with learning a bit about your background, in particular whether you were always into science, chasing animals in your backyard as a kid maybe?*

CC: *Oh no, absolutely not*! I always think that one of the reasons why I became a “*micro*” biologist was because I definitely was not into chasing animals. Even the deer in my backyard, I’d like them at the zoo, but not really in my backyard. As a first year graduate student, I had a very lucrative job paying $8/h working for a couple of immunologists to gather ascites fluid. At that time, $8/h was considered “big bucks”. When a mouse got sick, the littermates would hide him/her underneath the straw. So you can imagine that there were many traumatic moments when I discovered half-eaten body pieces and that really solidified which biological systems I would ultimately choose to work on, viruses, cells, etc. in other words, basic science…

*SK: And a virus that does not have a good animal model… So then, when did you first realize that you wanted to become a scientist?*

CC: Well, really I have to say that there was probably some priming done, as one of my earliest recollections is the encouragement received from an elementary school teacher that I had for the 4th, 5th and 6th grades. He used to give me old books that the library was getting rid of. One of them was called “The Book of Inventions”. It was tattered and had a brown hard cover. It was absolutely fascinating to read about the way people stumbled onto the things that they discovered. So, I would say there was some priming early on and then in high school that maybe got more focused.

*SK: So then, if you hadn’t gotten those books, what would you have become?*

CC: My family teases me because one of the things I love to do is to sing. But the thing about it is and the reason my family teases me is because *I can’t sing!* The other thing I love to do is to play the piano. And I bought one. But another thing I can’t do is play the piano. I definitely don’t have the time to practice. I keep promising them that “when I retire”, I am going to learn how to play the piano. But of course that’s ridiculous as I will probably have arthritic fingers!

*SK: That’s funny as I always also tell people that if I were not a scientist and had talent, I would become a singer! At least you didn’t say you would become a doctor.*

**Fig. 3:**
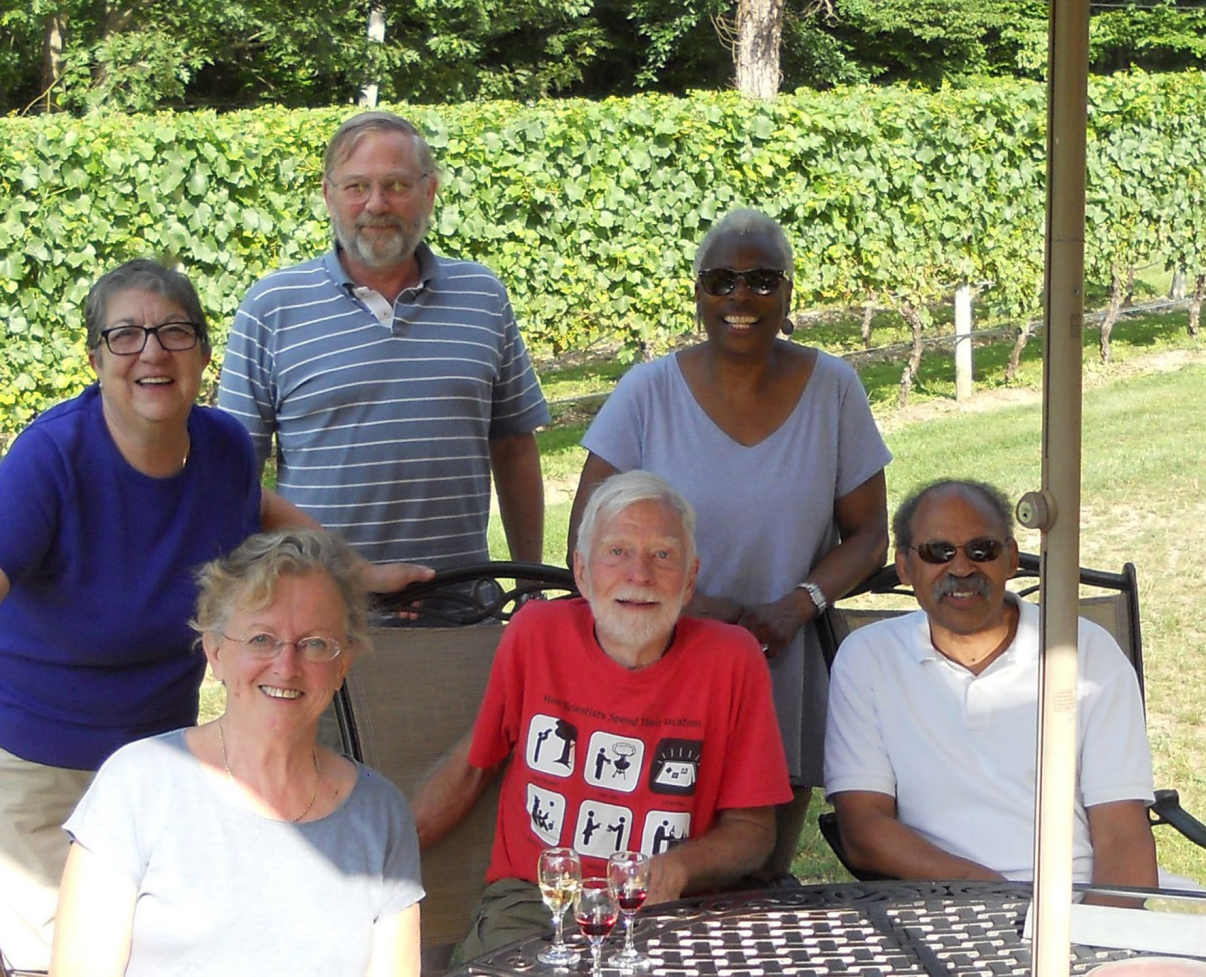
Dr. Carter and her Yale graduate school buddies in 2015

CC: *Oh, no way*! I mean, between the glamor gowns and the fun, what better profession would there be? As a matter of fact, my first year of graduate school was as much a fun year as it was an unusual year. It was the first time Yale had admitted a graduate class of 6 girls and only 1 boy as opposed to the other way round. So we definitely bonded and we are still friends today (Fig. 3). We put on a first year talent show where we substituted science lyrics for verses in *The Sound of Music*: We cast our department chair as the autocratic but kind Baron von Trapp and our faculty as the von Trapp children. It was great fun because it let me do all the things I like doing!

*SK: Funny you mention that! I have a group of super fun colleagues that I do similar things with at Wash U, including going to karaoke bars (of course pre-COVID). We recently did an ABBA dance at the retreat, sang Bad Project (the science-y version of Bad Romance by Lady Gaga) and recently did a talent show where I sang the American national anthem, nearly perfectly, while hoola-hooping! Do you have any other hobbies apart from playing or not playing the piano or any COVID related hobbies?*

CC: One is COVID itself, where there are super fascinating reads from every direction possible, mind boggling. And, of course, one of the things I really do find intriguing is all the parallels that you can begin to see between the various virus systems, no matter which one you’re working on, and coronaviruses. Another hobby, which is not so necessarily good, is cooking: We used to enjoy going out all the time, especially to New York City, but last year, one had to get creative inside your own kitchen. People who know me know that I also love to walk on the Stony Brook campus and I really love to walk on the Long Island beaches. I really couldn’t find good substitutes but that’s now beginning to change as summer arrives, thanks to the vaccine.

*SK: I always find this question difficult to answer, that is, how to balance work and life. I guess one of the most challenging moments in my career was when I was setting up my lab, trying to recruit and train people, while caring for a newborn. So, maybe you can give me some advice.*

CC: I sort of consider myself fortunate in that regard: I am married to someone who has 9 brothers and sisters. And since he is one of the eldest, who do you think ends up hosting birthdays, holidays, you name it! When there is a grant deadline coming up, especially the killer January 7th deadline that comes right after the holidays, one has to do a whole lot of planning, and organizing and delegating. So I needed to convince my husband, my son and my son’s friends to cheerfully take on much of the workload so as to give me time to think and to work on the grant application and the related activities.

*SK: You have been very successful in your career, but what was the biggest hurdle you had to overcome in launching it? Say during the first 5 years of becoming an assistant professor?*

**Fig. 4:**
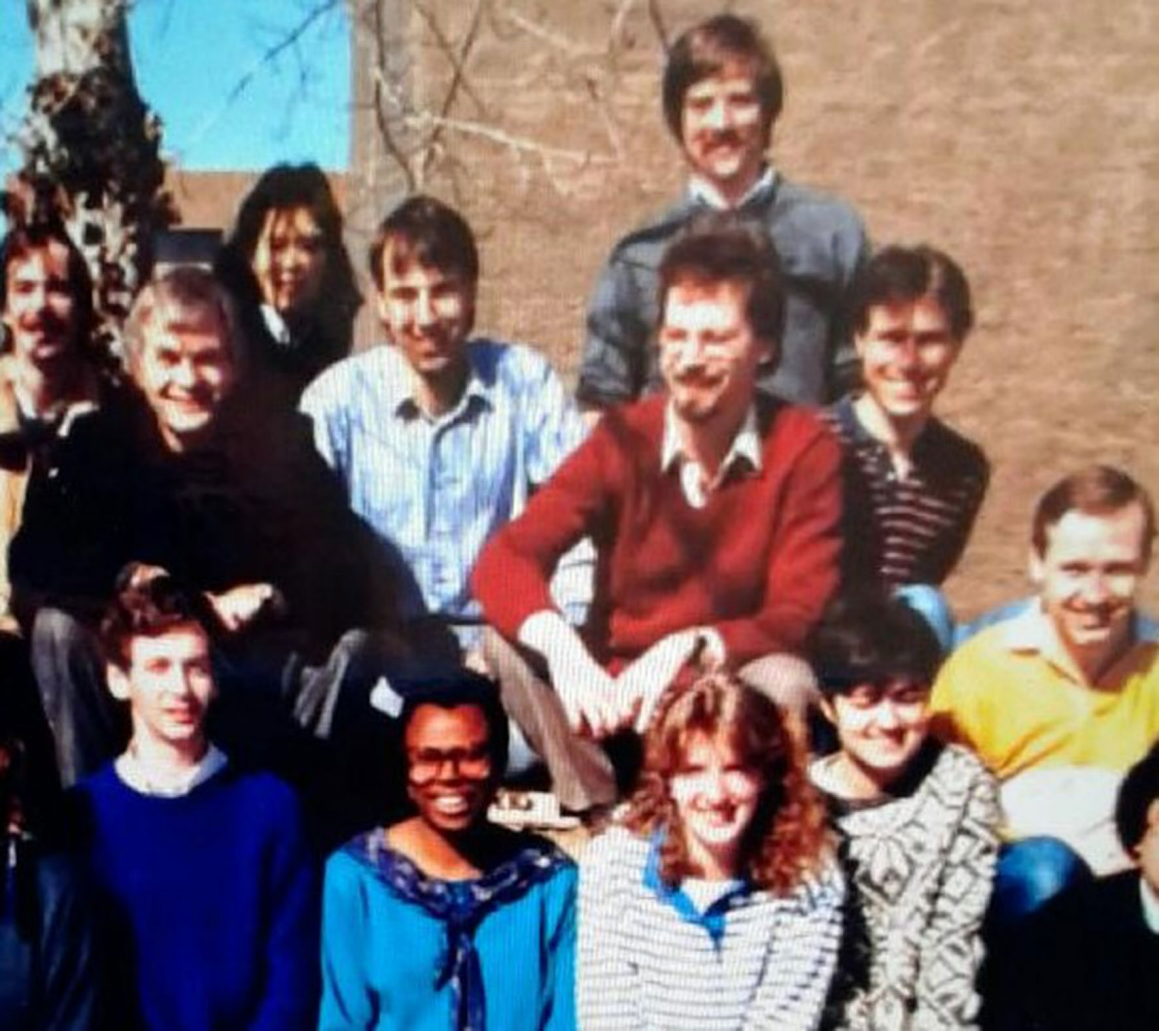
Dr. Carter and her colleagues. *1986–1991: SBU Carter—Wimmer Collaborators*

CC: I would say that the biggest hurdle was trying to learn how to write grants so that the reviewers would understand what question you are asking and why it is important. This is not trivial because in the first several years as an assistant professor you do know a whole lot but typically have only limited experience in writing and science communication. I found it very helpful to talk to fellow Assistant Professors outside my immediate field to obtain perspective on what I was thinking and to provide “big picture” understanding. I even attended meetings outside of virology, typically at my own expense. The older faculty in my department were fabulous, that’s for sure, but I also became really good friends with a person in a field outside my own, a yeast geneticist, with whom I could trade grants and papers. We gave each other really good feedback in that way regarding whether the message we were trying to convey was getting across, whether the hypothesis was well-grounded, and whether proposed approaches were feasible (Fig. 4).

*SK: You know, it’s interesting as I think, one of my hurdles has been recruiting and training the right people in the right way. How did things work out for you?*

CC: One of the reasons why I really like the PhD degree is because it is so personalized. I learned that I needed to understand how a student learns in order to guide them in a manner that is compatible with their intrinsic learning style. Many faculty believe there is only one right way to do things and you either do it their way or find another mentor. I think that just as scientists approach the same question using different strategies, training PhDs needs to be flexible to encompass the different ways in which students tackle how they learn. One of the experiences that gave me that notion was my being made Director of our Medical Microbiology course taught to medical and dental students very shortly after I became an Assistant Professor. The medical school was very, very sensitive to the fact that medical students pay a lot of money to go to medical school and they really wanted to support them so that they don’t flunk out. So the school encouraged course directors to accommodate diverse learning strategies. That made me believe that graduate student training should be viewed in exactly the same way. The reward for me was that I had to try to understand how the student was thinking about questions, which broadened my own perspective.

*SK: We heard about your motives getting into microbiology, but tell us more about how you got into virology, retrovirology and HIV over the years.*

CC: I started off studying bacteriophage because I found phage super fascinating but I really didn’t get along with my first advisor in graduate school. When I went to the chair of the department and told him that I had to get out of that lab*,* he considered me a heretic but permitted me to do so. So, for my second advisor, I chose a person who was 180 degrees different from the first one in just about every aspect. The most pertinent attributes were (1) he was a seasoned animal virologist—an epidemiologist who studied measles virus infections in indigenous populations in Brazil—who wanted to expand to basic virological research and (2) he was very game and adventuresome. He took me to meetings with him and he introduced me to his peers, who were senior faculty, exposing me to people whose names were in the textbooks of that time! And I think that he was very indulgent in letting me read the literature and figure out how to ask questions. One of the people he introduced me to, who was critical to my becoming an animal virologist, was Matthew Scharff at Albert Einstein University, now a member of the National Academy. Matty Scharff introduced the field to HeLa cells for cultivating animal viruses. As a postdoc, I was very interested in reoviruses because they were so unique with their double stranded RNA genome so I joined the laboratory of Aaron Shatkin. Fortunately for me, it was after I had achieved tenure to Associate Professor that the funding for reoviruses got very difficult because, although they were really interesting because of the double strandedness of the RNA, they did not cause any widespread disease. I knew it was time to switch fields when a NIH Program Officer to whom I had whined about funding said to me (and I quote), “*Carol, NIH is in the business of funding health, not interesting science*”. Lol.

*SK: Clearly coronaviruses!*

CC: Yes, clearly coronaviruses. But at that time, I actually decided to switch to, don’t laugh, SV40. After all, it’s a tumor virus. I spent a fabulous year in sabbatical—the only one that I’ve ever had the time to take. Anyway, I was eligible for a sabbatical and I took it. I had a fabulous, wonderful year where I learned all kinds of great techniques in the laboratory of Carol Prives at Columbia U. At the end of that sabbatical year, I naturally wanted to go to a meeting and hear what was going on with respect to that field. Imagine my surprise and chagrin when I see that there are no less than five hundred people at this meeting and that each one of them is, I swear, studying one of the bases in the viral genome! I said *oh my goodness, this was not necessarily the best choice for me*. So, I leave the meeting and I’m trying to figure out what should I do when, in fact, I start reading about this mysterious disease that’s afflicting people all over the world with illnesses caused by a variety of different pathogens that the immune system should be able to handle. The physicians and scientists had no clue what was going on. So now what I’ve done essentially is to bring you to the late 80 s and early 90 s when HIV started hitting the scene. So I immediately jumped into that and haven’t looked back since, because it’s been a great field to be a part of. And, just like coronavirus, it’s a field where in the early days any question that you ask is really important and helpful. And so, I think it was pretty fortuitous going to that meeting and finding so many people at it that I decided, *no*, the SV40 field was not where I wanted to be.

*SK: So now that NIH has spent thousands of dollars studying every nucleotide of the HIV genome, what excites you the most nowadays in the HIV biology and generally the retroviral field?*

Well, switching to HIV/AIDS certainly permitted me to combine health and interesting science! And, just like HIV was in the 90’s, coronavirus is now a field where any question that you ask is really important and can led to helpful new information. There’s no question that the field now knows a whole lot about HIV itself but HIV has been and continues to be a fabulous model for so many other pathogens, now including coronaviruses. And, just as coronaviruses are now, HIV is a major challenge, despite the fact that we’ve got a lot of tools. We know that the arsenal can easily disappear between drug resistance that could make it obsolete and the lack of a protective and eradicating vaccine.

*SK: Which brings me to my next question, which is that you must be very excited about capsid targeting compounds, long acting compounds, given years of your own research on understanding the molecular mechanisms of particle assembly.*

CC: Yes. The recent development of capsid targeting compounds is an impressive testimony to the translation of basic science into clinically deliverable goods. It’s been intriguing to witness, inspiring and humbling all at the same time. What’s humbling is the fact that it’s taken many decades and scientists looking at the question of capsid assembly from several different perspectives. Shortly after a very talented biochemist in my lab, Lorna Ehrlich, demonstrated that recombinant HIV-1 CA protein could oligomerize in vitro, I was fortunate enough to be asked by Michael Rossmann, an X-ray crystallographer who had solved the structure of so many plant and insect viruses by that time, to collaborate with him to solve its structure. X-ray crystallography did not permit more than a low resolution structure but fortunately, the effort was rescued by Mike Summers’ and Wes Sundquist’s NMR spectroscopy. Figuring out how the basic units of the capsid protein interact and how small molecules interfere has taken decades and incredible advances in imaging technology. What’s inspiring is how the field kept pushing to get these questions addressed, leading to the current achievement. HIV has certainly taught us that antiviral drug development is an invaluable therapeutic component.

*SK: We talked quite a bit about virology, retrovirology and science. So let’s get a little bit personal. The trainees always think that the PIs are like godlike creatures who never make mistakes. What was your biggest mistake in the lab that you don’t want anybody to hear about?*

CC: Oh, yes. Rabbit holes!!! Mother Nature is so tricky. You make some finding or you have some idea… And the idea is, as far as you’re concerned, so obvious, so simple, so fabulous. How come other people haven’t published this already? *Well, often times, that’s because it is a rabbit hole!* In this category falls the couple of years we spent trying to establish the Ty retrotransposon as a model system so that we could study HIV-1 in yeast. It seemed like such a good idea at the time.

*SK: And so imagine this was a project for a graduate student and obviously the graduate student might have some problems with it. So what would you recommend to a graduate student who wants to pursue academic research?*

CC: I would recommend that they base the decision on what they know about themselves and their comfort level in dealing with challenges within and outside their control. I would provide as an example the story of Beth Agresta, a wonderful graduate student in my lab around two decades ago. She was, in fact, the student who conducted a yeast-two hybrid library screen, out of which we picked up Tsg101 as a cellular protein that was interacting with HIV-1 Gag. She also fished out cyclophilin. When Beth looked for the protein sequences in the protein database she found only cyclophilin and we realized that Tsg101 was unknown. She asked herself a critical question: Do I want to do my thesis research on a protein about which nothing is known or should I select the one that is known so that I can relate my findings to those of others? I think a graduate student who wants to pursue academic research needs to feel comfortable with the several unknowns that will be encountered when one is in unchartered territory. Beth knew precisely what she wanted to be doing in 10 years from that time and she made her decision accordingly.

*SK: So what is your advice?*

CC: My advice is to decide what you want in terms of your long term goals. Students who are “lab rats”, *i.e*., who never leave the lab or start early and leave late, love working at the bench and tinkering are probably more suited to academic research than students who envision completing their studies in a pre-defined number of years. The latter may find defining new systems or developing new methodologies to address a question frustrating if the process presents challenges. I’ve been fortunate to have wonderful undergrads, graduate students and postdocs who knew themselves well and had defined their goals realistically, so that is great. Setbacks and failures can teach one a lot but only if one is patient enough to accept them and learn from them.

*SK: That’s what I tell my son.*

CC: Yes, me too.

*SK: I’m going to ask a final question of you, since you’ve worked on multiple viruses and different aspects of virus replication. What is your favorite virus and why?*

CC: I have to say that HIV has definitely supplanted the reovirus of my early days. And it’s quite competitive with SARS-CoV-2 although that virus is very fascinating. In general, SARS-CoV-2 fascinates me the most in the aspects with which it differs from its relatives, the coronaviruses that emerged recently but did not cause pandemics. Not surprisingly, I am fascinated by its proteases, its nucleocapsid protein and how it traffics its protein to the exit site. All of those are aspects that HIV can inform about. So HIV and other retroviruses are still definitely my favorite viruses because they taught us so much and have potential to continue to do that.

*SK: That is a great ending for a Retrovirology article! Dr. Carter this was a wonderful conversation and the first in its series. Thank you for your time and I look forward to continuing these conversations when we meet in person!*

